# Principles of Physiology; Designed for the Use of Schools, Academies, Colleges, and the General Reader

**Published:** 1855-01

**Authors:** 


ART. VIII.—*Principles of Physiology; designed for the use of Schools, Academies, Colleges, and the General Reader. Comprising a familiar explanation of the Structure and Function of the Organs of Man, illustrated by comparative reference to those of the inferior animals. Also an Essay on the Preservation of Health. With fourteen quarto plates and over eighty engravings on wood, making in all nearly* 200 *figures.* By J. C. COMSTOCK and B. N. COMINGS, M. D. New York: Samuel S. & WILLIAM WOOD. 1855.

This comprehensive title gives a general idea of the scope and purposes of the work. Very many text-books of physiology for schools which have passed under our notice, have been so imperfectly arranged, and their ideas so erroneously or incorrectly expressed, that they must necessarily have been rather a detriment than a benefit to the scholar.

So far as we have examined the text of the volume under notice, it is more free from these objections than most of its predecessors. Attempting to give only the settled conclusions of established physiological doctrine, it thus avoids hypothesis and the crude notions of diet, exercise, and bathing, which are found in some of our school books, and which are undoubtedly productive of serious injury to the health of youth.

The outside appearance and inward illustration of Messrs. Comstock and Comings' Physiology, deserve some notice. It is printed in large quarto form, the text corresponding to the plates. Interspersed among the text are a large number of clever wood engravings, mostly microscopic views of different tissues.

Aside from these are fourteen large quarto plates. These are colored lithographs of very superior execution, illustrating the principal and most important parts of human anatomy, accompanied occasionally by views from comparative anatomy, or by microscopic illustrations. We have never seen anything neater in their way than these quarto plates. Artistically they are very neatly executed, while anatomically they are entirely correct. So far as plates can teach they are excellent.

We should be glad to see this book substituted for some of the coarsely prepared and incorrect treatises now in circulation. H.

